# Low Rate of Germline Investigation for Variants of Suspected Germline Origin Detected During the Diagnostic Work‐Up of Myeloid Neoplasms

**DOI:** 10.1002/jha2.70206

**Published:** 2025-12-16

**Authors:** Ahmed Al‐Djaber, Arielle Munters, Jörg Cammenga, Tatjana Pandzic, Rose‐Marie Amini, Claes Ladenvall, Thale Kristin Olsen, Panagiotis Baliakas

**Affiliations:** ^1^ Department of Immunology, Genetics and Pathology Uppsala University Uppsala Sweden; ^2^ Clinical Genomics Uppsala, Science for Life Laboratory Uppsala University Uppsala Sweden; ^3^ Department of Hematology, Oncology and Radiation Physics Skåne University Hospital Lund Sweden; ^4^ Division of Molecular Medicine and Gene Therapy Lund University Lund Sweden

**Keywords:** genetic analysis, genetic predisposition, molecular diagnostic techniques, myeloid neoplasm

## Abstract

**Background:**

Genomic profiling of patients with myeloid neoplasms (MN) may detect variants with high variant allele frequency (VAF), indicating potential germline origin. All current guidelines recommend further investigation of such variants.

**Objective:**

To define the proportion of variants with suspected germline origin that prompted germline investigation in patients evaluated for MN in the clinical setting.

**Methods:**

We included 738 patients investigated for MN in Mid‐Sweden between May 2020 and January 2023 with available sequencing data in genes of interest (GOI) according to the Nordic and other international guidelines.

**Results:**

In total, 90 patients (12%) carried pathogenic/likely pathogenic (P/LP) variants in GOI according to the Nordic guidelines, with *TP53*, *RUNX1*, and *NRAS* being the most common (*n* = 37, *n* = 27, and *n* = 9, respectively). The potential germline origin was investigated in 16/90 (18%) patients. Two patients (2/16, 12.5%) with germline variants were identified (*DDX41* and *RUNX1*, *n* = 1, respectively). In patients treated with allogeneic stem‐cells transplantation (*n* = 15) the investigation rate was higher (7/15, 47%, *p* < 0.005) compared to the non‐transplanted ones. Applying the European Leukemia Net (ELN) guidelines the number of patients with variants in GOI increased up to 177 mainly due to the inclusion of *TET2*, while application of other international guidelines leads to similar results with the Nordic ones.

**Conclusion:**

We report a low investigation rate for variants of suspected germline origin detected during the genomic profiling of patients investigated for MN in the clinical setting. Our study underscores the need for further awareness regarding germline investigation for hematological malignancies.

**Trial Registration**: The authors have confirmed clinical trial registration is not needed for this submission.

## 1 Introduction

Traditionally, myeloid neoplasms (MN) were perceived as sporadic, driven mainly by somatic alterations, with only a few exceptions associated with rare hereditary conditions such as Down syndrome, Fanconi anemia, and telomere biology disorders (TBD) [1, 2]. The introduction of high‐throughput sequencing (HTS) has elucidated the genetic landscape of MN revealing disease‐specific aberrations that impact on diagnosis, risk stratification, treatment, and monitoring of response. In addition, the incorporation of HTS in routine diagnostics has provided unprecedented insight into the etiology of these disorders, unveiling a previously underestimated role of germline predisposition [1, 3, 4].

Recent studies suggest that 5%–15% of all MN, mainly acute myeloid leukemia (AML) and myelodysplastic syndrome (MDS) have an underlying hereditary component [5, 6]. Identifying such germline variants has direct implications in the clinical management, such as the choice of donor and/or condition regimen in the context of allogeneic hematopoietic stem cell transplantation (allo‐HSCT). Moreover, specific germline conditions such as *DDX41* related‐MN, the most common germline form of MN, seem to constitute distinct clinical entities with unique clinical behavior that overcomes the known prognostic and predictive algorithms [7, 8, 9, 10]. In addition, identification of germline variants may assist clinicians in risk assessment and tailoring surveillance programs for patients and their unaffected family members [11, 12].

For the time being, there is no consensus on whom to test for germline predisposition among patients with MN. However, all published international guidelines concede that variants of suspected germline origin detected during the diagnostic work‐up of MN should be further investigated [13, 14, 15, 16] independently of family history (Table 1). This recommendation is based on the current praxis with the application of tumor‐only sequencing of comprehensive gene‐panels at the diagnosis of MN, panels including genes both somatically mutated in the conditions in focus but also associated with germline predisposition [17].

**TABLE 1:** List of genes included in the high‐throughput sequencing (HTS) based myeloid panel used at Uppsala University Hospital that are associated with germline predisposition to myeloid neoplasms, according to the different guidelines. Green color indicates that guidelines suggest an association with germline predisposition. Conversely, red color indicates that the guideline does not endorse the gene as part of a germline investigation.

Gene of interest (GOI)	Nordic	ELN	ICC	WHO	NCCN	UK
*ANKRD26*						
*CEPBA*						
*CSF3R*						
*DDX41*						
*ETV6*						
*GATA1*						
*GATA2*						
*JAK2* ^*^						
*MPL*						
*NPM1* ^*^						
*RASopathy related genes* ^**^						
*RUNX1*						
*SAMD9*						
*SAMD9L*						
*TET2*						
*TP53*						
*TERC*						
*TERT*						

* Somatic hotspot variant excluded.

** CBL, KRAS, NF1, NRAS, PTPN11.

Some of these genes are implicated in syndromes where the risk for MN is only one of the many clinical manifestations as in the case of RASopathies, *GATA2*‐related syndrome or TBD, or general predisposition for cancer (Li‐Fraumeni) [18, 19, 20, 21]. Other genes are associated with cytopenia preceding the MN such as *RUNX1* or *ETV6* [22, 23]. The list of these candidate genes for potential germline investigation differs slightly across the various recommendations, with the most comprehensive being the one proposed by the European Leukemia Net (ELN), probably due to fact that they are among the latest published (Table 1).

Regarding germline predisposition, Sweden follows, since 2019, the Nordic recommendations published by the Nordic MDS study group. Similar to other, the Nordic guidelines suggest further investigation of any pathogenic/likely pathogenic (P/LP) variant in genes linked to germline predisposition detected during the diagnostic work‐up of MN, if VAF is indicative of germline origin [4].

The aim of the present study is to evaluate whether the detection of a variant of suspected germline origin during the genetic profiling of MN, initiated germline investigation as recommended by the Nordic guidelines, or if there is still room for improvement regarding the implementation of the recommendations on germline investigation for MN in the clinical setting. This study also wants to raise awareness of the high number of patients that should be investigated for germline predisposition, an approach that demands broad expertise and resources with many implicated health professionals as hematologists, genetic counsellors, and clinical geneticists.

## 2 Methods

### 2.1 Study Design

This retrospective observational study included patients (excluding children < 18 year) with suspected MN who underwent HTS myeloid panel testing at the Uppsala University Hospital, Sweden, between May 2020 and January 2023. Patients were referred from the healthcare region of Mid‐Sweden, which has a population of 2.1 million.

### 2.2 Data Collection and Analysis

For detailed information about sequencing, variant calling and interpretation see Supporting Information (S1 and S2). We focused on routinely reported variants in the genes of interest with VAF ≥ 40%. Somatic hotspot variant in *JAK2* (e.g., V617F) and *NPM1* (mut A–D) were excluded, since such variants alone are not expected to motivate further germline investigation according to Nordic guidelines. Sub‐analysis with variants with VAF 30%–40% was also performed. Variants absent in follow‐up samples were considered as somatic. Germline investigation also includes the analysis of CD3 positive cells in non‐T‐cells related malignancies, as well as analysis of DNA extracted from cultured fibroblasts. HTS data, clinical data, demographic information, and germline investigation status was extracted from electronic medical records and local databases. The proportion of patients that carry a suspected germline variant warranting further targeted analysis was defined depending on the applied guidelines (Nordic, ELN, ICC, WHO, NCCN, UK), focusing on the Nordic guidelines.

### 2.3 Statistics

Statistical analyses were performed using R (ver. 4.5.0). McNemar's test was used to compare overlapping subsets of patients according to different guidelines within the same cohort, while Fisher's exact test was applied for independent comparisons. A *p*‐value < 0.05 was considered statically significant. Statistical measures, including mean, median, and standard deviation, were calculated to describe distributions. Figures and graphical representations were created using GraphPad Prism version 10.6.1.

## 3 Results

**FIGURE 1:**
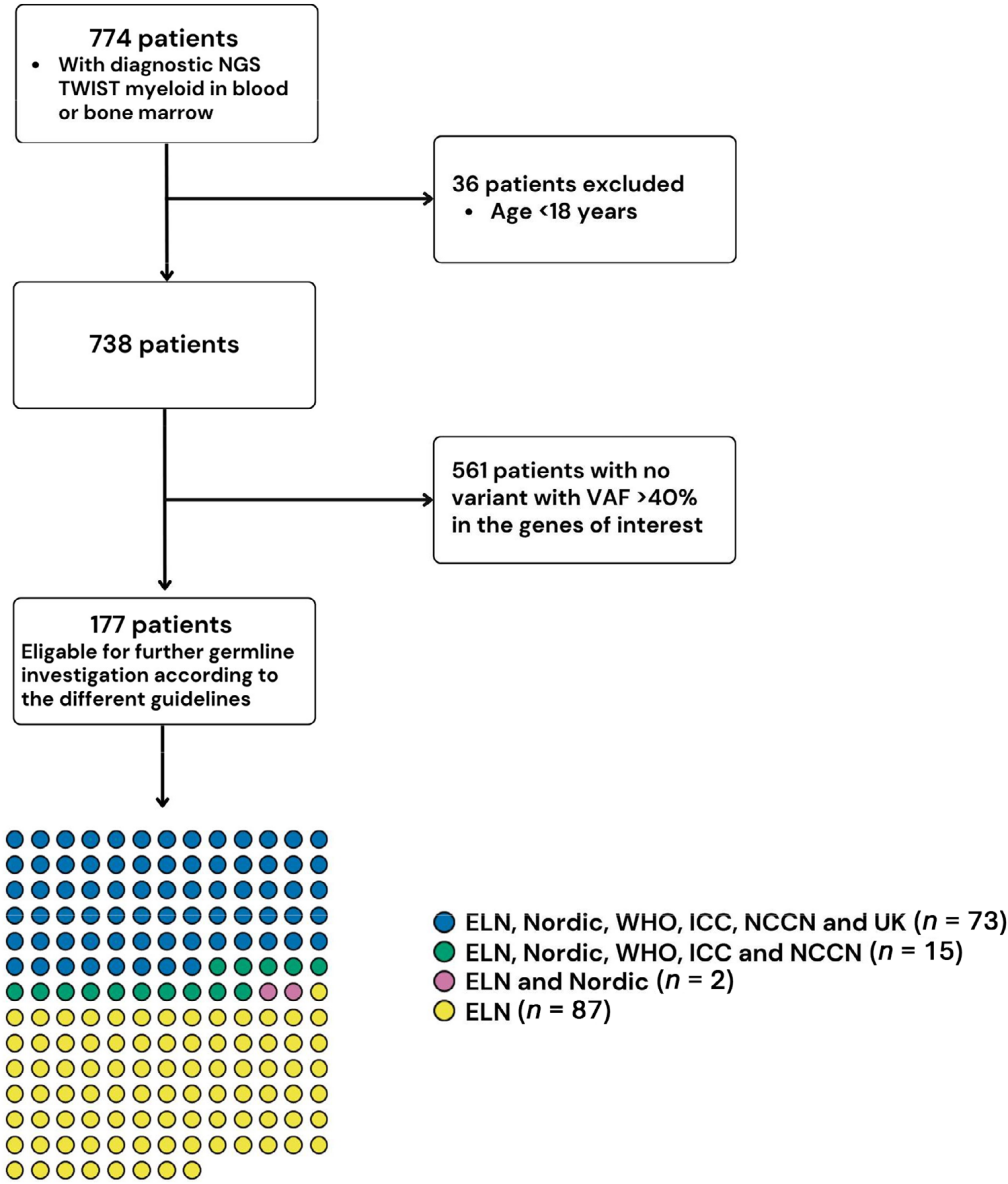
Inclusion‐exclusion flow chart. According to the Nordic guidelines 90 of 738 patients tested with high‐throughput sequencing (HTS) based myeloid panel was eligible for further germline investigation. With TET2 and MPL included in the ELN‐recommendations an addition of 84 patients had a variant in at least one gene of interest gene with a variant allele frequency ≥ 40% leading to a total of 177 patients.

HTS with the diagnostic myeloid panel described above, was performed in 774 patients during the study period. Thirty‐six patients were excluded due to low age (< 18 years) (Figure 1). A total of 738 patients were finally included in the study with a mean age of 66.7 years and a slight overrepresentation of males (Table 2). The most common indications for testing were suspected/confirmed MDS (61%), followed by suspected/confirmed AML (23%), MPN (9%), MPN/MDS (3%), and other hematological malignancies (3%) (Table 2). Diagnostic testing with HTS was analyzed in either bone‐morrow (86%) or in blood (14%).

**TABLE 2:** Descriptive analysis of the study cohorts. Eligible patients carry one or more pathogenic/likely pathogenic (P/LP) variants in a gene of interest according to the guideline. The percentage for each variable in parentheses refers to the proportion of patients within the cohort.

Variables	Sub	Eligible patients Nordic (*n *= 90)	Eligible patients ELN (*n *= 177)	Eligible patients WHO/ICC/NCCN (*n* = 88)	Eligible patients UK (*n *= 7)	All patients (*n* = 738)
**Gender**	**Male**	53 (59%)	107 (60%)	52 (59%)	44 (60%)	412 (56%)
	**Female**	37 (41%)	70 (40%)	36 (41%)	29 (40%)	326 (44%)
**Age**	**mean ± SD (median)**	69.5 ± 11.4 (71)	72.2 ± 10.5 (74)	69.5 ± 11.5 (72)	69.7 ± 11.6 (72)	66.7 ± 15.1 (71)
	**< 50**	5 (6%)	6 (3%)	5 (6%)	4 (5%)	100 (14%)
	**50–70**	35 (39%)	52 (29%)	33 (38%)	27 (37%)	257 (35%)
	**> 70**	50 (56%)	119 (67%)	50 (57%)	42 (58%)	381 (52%)
**Suspected disease**	**Acute leukemia**	48 (53%)	66 (37%)	46 (52%)	40 (55%)	169 (23%)
	**MDS**	28 (31%)	82 (46%)	28 (32%)	24 (33%)	451 (61%)
	**MPN/MDS**	7 (8%)	14 (8%)	7 (8%)	5 (7%)	24 (3%)
	**MPN**	7 (8%)	15 (8%)	7 (8%)	4 (5%)	69 (9%)
	**Other**	0 (0%)	0 (0%)	0 (0%)	0 (0%)	25 (3%)
**# P/LP‐variants in a gene of interest**		101	207	98	76	—

**FIGURE 2:**
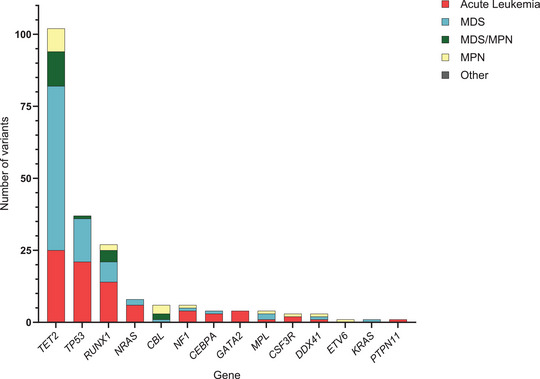
Total number of pathogenic and likely pathogenic variants with variant allele frequency ≥ 40%, note that one patient might have several unique variants.

With the application of the Nordic criteria and following the cut‐off of 40% VAF, a total of 101 P/LP variants in 90 patients were identified, with *TP53*, *RUNX1*, and *NRAS* being the most implicated genes (*n* = 37, *n* = 27, and *n* = 9 patients, respectively). The median age was 69.5 years (± 11.4) with five patients (6%) being younger than 50 years and 50 patients (56%) above 70 years at the time of investigation (Figure 2, Table 2). Suspected/confirmed acute leukemia was the most common indication (*n* = 48 patients, 53%) in patients with P/LP variants, followed by MDS (*n* = 28, 31%), MDS/MPN (*n* = 7, 8%), and MPN (*n* = 7, 8%). While *TP53* predominated in our cohort, only one patient was detected with a *TP53* variant in the MDS/MPN and MPN sub‐group, where *RUNX1* (*n* = 6, 43%) and the RASopathies‐related genes, *CBL* (*n* = 5, 36%) and *NF1* (*n* = 1, 7%) were most prevalent (Figure 2, Table 2).

Germline investigation was most common among patients with the indications of acute leukemia (11/48; 23%) followed by MDS (4/28; 14%), MPN (1/7; 14%), and MDS/MPN (0/7; 0%). Comparing the age groups, the highest investigation rate was observed in patients aged 50–70 years (11/35; 31%), followed by < 50 years (1/5; 20%) and > 70 years (4/50; 8%) (See Supporting Information S3).

Following the ELN criteria the number of P/LP variants doubled reaching up to 207 variants, primarily due to 102 detected variants in *TET2* (See Supporting Information S4). Resulting in an increase in individuals (*n* = 177) meeting the eligibility criteria (*p* < 0.001 vs. Nordic guidelines). The median age was 72.2 (± 10.5) with six patients (3%) below 50 and 119 patients (67%) above 70 years at the time of investigation. Applications of the WHO, ICC, and NCCN recommendations resulted in 98 variants in 88 patients, similar to the Nordic guidelines, while the UK‐guidelines resulted in 76 variants in 73 patients (*p* < 0.001 vs. Nordic guidelines), all with similar characteristics to the ones observed with the application of the Nordic guidelines (see Table 2).

**FIGURE 3:**
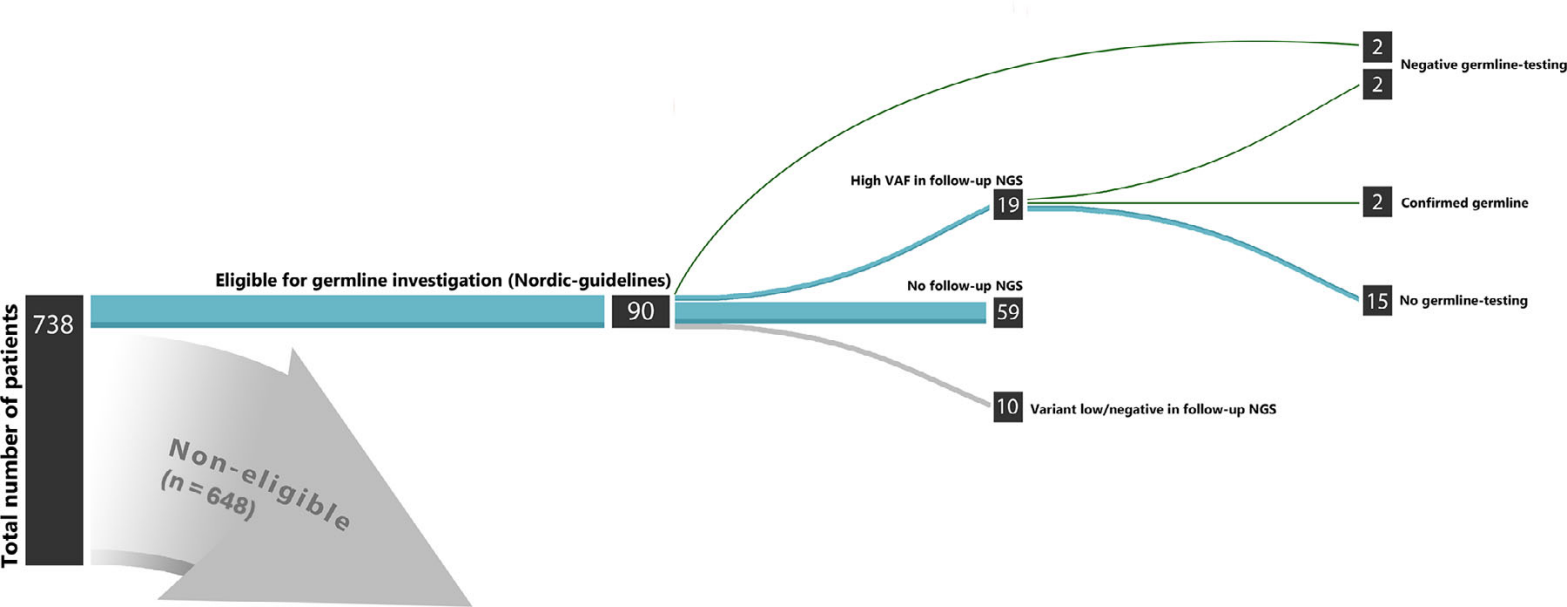
Investigation flow‐chart for the patients (*n* = 90) with variant allele frequency ≥ 40% in a gene of interest according to the Nordic guidelines. Blue color marks patients eligible for further germline investigation, gray means that the patients do not meet the criteria for further investigation. Green indicates complete investigation with germline verification.

Among the 90 patients with eligible variants for further germline investigation according to the Nordic guidelines, 29 patients underwent MRD‐assessment with a follow‐up HTS. In 10 of these cases the frequency of the variant had decreased significantly or was undetectable, indicating that the variants were of somatic origin. In 19 patients, a high VAF of the variant remained, warranting further germline investigation (Figure 3). Four of these patients proceeded with germline verification and two were confirmed to be germline. In addition, two patients underwent direct germline verification without any prior MRD follow‐up. Overall, the presence of a germline variant was investigated in 16 (18%) patients, while in the remaining 74 (82%) patients no germline test was performed. Analysis of the six cases that underwent germline verification was either performed on CD3^+^ cells from blood/bone marrow or DNA extracted from fibroblasts (*n* = 3 and *n* = 3, respectively) with variants in *RUNX1*, *DDX41*, *KRAS*, *TP53, and GATA2* (Table 3). Two patients were confirmed to be carriers of a germline variant in a gene of interest (*DDX41* and *RUNX1* respectively, Table 3).

**TABLE 3:** **S**ummary of patients who underwent a complete germline investigation, including analysis of normal tissue to verify suspected germline variants. The table presents the verification method, identified variants, and results of the investigation.

Case	Age	Suspected disease	Sample	Verification method	Gene	Type	Result
67	> 70	MPN	Fibroblasts	NGS‐targeted analysis	*DDX41*	Splice‐variant	Confirmed germline
159	< 50	MDS	Blood (CD3^+^)	Exome sequencing	*KRAS*	Missense	Negative
187	50–70	Acute leukemia	Fibroblasts	Exome sequencing	*TP53*	Missense	Negative
242	50–70	Acute leukemia	Bone marrow (CD3^+^)	NGS‐targeted analysis	*GATA2*	Missense	Negative
314	50–70	Acute leukemia	Fibroblasts	NGS‐targeted analysis	*RUNX1*	Truncating	Confirmed germline
					*RUNX1*	Missense	Negative
580	50–70	Acute leukemia	Blood (CD3^+^)	NGS‐targeted analysis	*GATA2*	Truncating	Negative
					*RUNX1*	Missense	Negative

**FIGURE 4:**
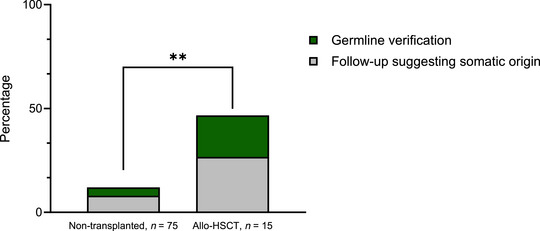
The percentage of completed investigation following Nordic guidelines for patients treated with allo‐HSCT and non‐transplanted patients (Fisher's exact test, *p* = 0.0043). Completed investigation is defined as germline verification or follow‐up suggesting somatic origin (i.e., low variant allele frequency in MRD‐testing prior to allo‐HSCT).

Of the 90 patients eligible for germline investigation according to the Nordic guidelines, 15 patients (17%) underwent allo‐HSCT, 2 of which were transplanted with a related donor. In 7 of those 15 patients (47%), a measurable residual (MRD) disease assessment was performed after the administration of treatment and before allo‐HSCT, with four patients exhibiting lower VAF in the variants of interest, indicating that these variants were of somatic origin (Figure 4). Three of those four patients had a single variant in a gene of interest *TP53* (*n* = 2) and *RUNX1* (*n *= 1) while one patient had both a *TP53* and an *NRAS* variant. The mean age of these patients was 66.5 (SD 5.1) years, while the referral reason was acute leukemia (*n* = 2) and MDS (*n* = 2). All four patients were transplanted with an unrelated donor. In the remaining three patients with MRD data, the variants of interest showed stable high VAFs. One patient (age: 56 years) with AML carried two variants in *RUNX1*, both with VAF over 40%. Germline investigation with targeted analysis of fibroblast cells verified that one of the *RUNX1*‐variants was of germline origin. The patient was later transplanted with an unrelated donor. The second patient (age: 62 years) was investigated for two variants in *RUNX1* and *GATA2* with a targeted analysis of CD3^+^ cells in blood, with a negative result indicating that the variants were somatic. Finally, the third patient (age: 68 years) who had a *CBL*‐variant exhibiting high VAF in follow‐up analysis underwent an allo‐HSCT with a related donor, without germline investigating of the variant of interest.

Among the eight patients treated with allo‐HSCT with no available MRD data, one of the patients underwent direct germline verification (negative for *GATA2*) without any prior MRD follow‐up and was later transplanted with an unrelated donor. Five other patients with variants in *TP53* (*n* = 3), *NF1* (*n* = 2), *RUNX1* (*n* = 1), and *NRAS* (*n* = 1) received a graft from an unrelated donor, while one patient with AML and a debut age at 46 years, with a *RUNX1*‐variant was transplanted with a related donor. One patient was transplanted in another region; hence, the donor's relationship to the patient is unknown. No germline investigations with targeted gene analysis were performed in these seven patients.

**FIGURE 5:**
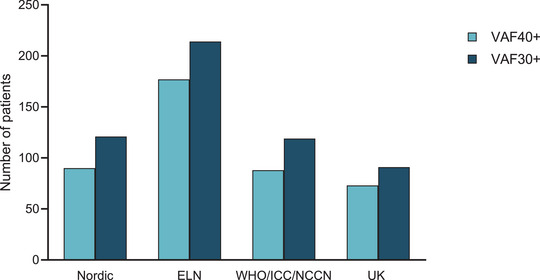
Eligibility for germline investigations in our cohort (*n* = 738) according to 30% and 40% variant allele frequency cut‐offs in the different guidelines.

We next evaluated the proportion of eligible patients for germline investigation according to the Nordic guidelines by applying a VAF cut‐off of 30%. The total number of patients increased to 121 with 63 of those (52%) being investigated due to suspicion of acute leukemia, with the spectrum of implicated genes being similar to the one observed when we applied the 40% cut‐off. Following the ELN criteria and 30% cut‐off resulted in the identification of 214 patients eligible for germline investigation mainly due to the presence of *TET2* variants as described above (See Supporting Information S4). Application of WHO, ICC, and NCCN lead to 119 patients similarly to the Nordic guidelines, while application of the UK guidelines led to 91 eligible patients (Figure 5).

## 4 Discussion

The recognition of the potential germline background in a significant proportion of patients with MN is probably one of the most important developments in the field of hematology during the last decade. The shift from the older dogma that hereditary MN is extremely rare to the current evidence‐based concept that up to 5%–15% of MN occur due to germline variants has revolutionized the management of these patients, especially in terms of the diagnostic procedures.

Several guidelines and consensus statements have recently been published with the aim of providing advice on how to implement this new knowledge in the clinical setting. Yet many challenges remain. As germline investigation cannot be offered to all patients with MN, several criteria motivating germline testing have been proposed. One that is widely accepted is the presence of P/LP variants detected during the somatic profiling, where the VAF suggests a germline origin, independently of the family history. This criterion is included in all international guidelines, and therefore it would be expected that it is followed in everyday clinical setting, especially in Sweden that has been a pioneer in the field of germline investigation. The findings of our study indicate, however, that clinical reality may differ from what is expected according to the guidelines.

We report a rather low investigation rate of potentially germline variants detected during the somatic profiling of suspected or confirmed MN in the whole cohort (16/90; 18%), when applying the Nordic guidelines. Interestingly, the persistence of the variants of interest in a follow‐up sample and with a high VAF, did not either result in further germline investigation for the great majority of the cases (Figures 3 and 4). As expected, the number of patients that ought to be investigated is even higher when we apply the ELN guidelines, as ELN proposes a longer list of candidate genes compared to Nordic but also other international guidelines. This low investigation rate could be in reality even lower, if we take into consideration the fact that even in families with MN and strong suspicion of germline investigation the potential underlying genetic is still unknown despite comprehensive genomic characterization [24]. Moreover, novel candidate genes are continuously reported in the literature [25]. Finally, not all genes implicated in germline predisposition, i.e., *ERCC6L2*, were included in the panel at the time of the performance of the study [26].

One may argue that the majority of the variants of interest i.e., *TP53* and *RUNX1*, when germline, have a rather characteristic spectrum of clinical features, which most probably was absent in the respective patients and therefore further germline investigation may not be considered necessary. Similarly, *TET2* variants, which account for the majority of additional eligible patients when applying the ELN criteria, are extremely rare and associated with specific clinical phenotypes. Indeed, the clinical or the family history may were non‐indicative of a germline background for the patients included in our study. With that been said, we and others have encountered patients with the variants above who did not exhibit the expected clinical spectrum or carried variants that were de novo and therefore the family history was not suggestive of any germline condition [22, 24, 27].

Another parameter that may explain the low investigation rate of variants of interest is that we report the data from the perspective of the diagnostic laboratory without having complete knowledge of all the clinical information. This is a major limitation of our study, since we ignore whether the patients that should have been further investigated decided actively to refrain from any germline testing. Based however, on our experience the latest 8 years during which more than 400 patients have been referred to our clinic with the suspicion of hereditary hematological malignancies, when patients with MN are asked whether they are interested in germline investigation tend to participate with great will. Moreover, in our cohort we have included even cases with suspected MN being investigated even due to unexplained cytopenia, which could also explain the rather low rate of P/LP *DDX41* variants.

Among the patients that underwent allo‐HSCT the proportion of the variants that were thoroughly investigated was higher (7/15, 47%) suggesting that in the context of allo‐HSCT there is greater interest of the potential germline background, since the identification of such variants directly impacts on the clinical decision related to the choice of donor, the conditioning or the follow‐up of the patient. Moreover, many reports on donor‐derived leukemia underscore the importance of investigating any potential germline variant before proceeding with allo‐HSCT, especially a family donor [28, 29, 30]. With that been said, we identified a case with a suspected variant of germline origin who despite being transplanted with a sibling donor, no germline investigation was carried out.

Our findings are in line with the common observation that clinical reality is not always in line with the current guidelines and does not comprise with the golden standard. This discordance may arise from the lack of resources, time or by the fact that the proposed guidelines may failed to reach out to the real clinical world and are considered outdated, not well motivated or unfeasible [31, 32, 33, 34]. Especially in the field of hematology, the concept of “a germline disease” is still not accepted by the whole community. Cost may also be of importance. In Sweden, however, where the cost of germline test is fully re‐reimbursed, one would expect higher investigation rate for variants of potential germline origin.

Focusing on our geographical healthcare region, with more than 7 administrative regions and 23 regional hospitals to serve, implementing novel diagnostic pipelines is a major challenge. Therefore, the structure of the Swedish system favoring de‐centralization may be another explanation for the lower investigation of potential germline variants. This is also supported by the observation that the investigation rate of variants of interest at least for the patients that underwent allo‐HSCT was higher compared to the non‐transplanted patients. Of note, all these patients, except one, were transplanted in the same university hospital.

In conclusion, we report a discordance between the guidelines that suggest further investigation of potential germline variants detected during the somatic work‐up of patients with MN and the clinical reality where a low number of such variants were actually investigated. Our results highlight the need for further awareness regarding the germline background in hematology and underscore that there is still room for improvement in this area. Further actions, especially aiming in educating the involved health professionals as well trying to overcome caveats of the health system may promote the establishment of germline investigation in patients with MN.

## Author Contributions

**Ahmed Al‐Djaber**: writing – original draft, visualization, methodology, formal analysis, project administration, data curation, investigation. **Arielle Munters**: writing – review and editing, data curation. **Jörg Cammenga**: writing – review and editing. **Tatjana Pandzic**: writing – review and editing, methodology. **Rose‐Marie Amini**: writing – review and editing. **Claes Ladenvall**: writing – review and editing, methodology. **Thale Kristin Olsen**: writing – review and editing, visualization, supervision. **Panagiotis Baliakas**: writing – review and editing, methodology, supervision, funding acquisition.

## Funding

A.A.D. received support from ALF Kansliet Uppsala (LUL‐990593). P.B. received support from ALF Kansliet Uppsala (ALF‐990591, ALF‐1002575, ALF‐991381, Swedish Society of Medicine (SLS‐961271), Lions CancerFonden (2022‐1050146, 2023‐1050180), Vleugels stiftelse (2020‐01), Jeansson Stiftelser (2023), and Swedish Cancer Society (23 2745 Pj, Projekt). J.C. received funding from the Swedish Cancer Foundation (Cancerfonden, 21 1823 Pj‐BF1) and the Swedish Childhood Cancer Foundation (Barncancerfonden, PR 2022‐0135, PR2020‐0128). T.K.O. received funding from the Swedish Childhood Cancer Foundation (Barncancerfonden, TJ2021‐0010).

## Ethics Statement

This study was approved by the local Regional Ethical Committee (Dnr 2023‐00987‐01) in accordance with the declaration of Helsinki.

## Consent

The authors have nothing to report.

## Conflicts of Interest

The authors declare no conflicts of interest.

## Supporting information



Supporting File 1

Supporting File 1

Supporting File 1

Supporting File 1

## Data Availability

The data that support the findings of this study are available on request from the corresponding author. The data are not publicly available due to privacy or ethical restrictions.
