# Supplementary material for: Low Rate of Germline Investigation for Variants of Suspected Germline Origin Detected During the Diagnostic Work‐Up of Myeloid Neoplasms
Source: EJHaem. 2025 Dec 16;6(6):e70206. doi: 10.1002/jha2.70206 (PMC12707176; doi:10.1002/jha2.70206)
Supplement: Supplementary file 2 — Supporting File 1 [file JHA2-6-e70206-s003.docx]

**Methods**

**DNA sequencing**

Genomic DNA was extracted from bone marrow or whole blood using the EZ1 DNA Blood Kit (QIAGEN) and was quantified using the Qubit dsDNA BR Assay kit on the Qubit 2.0 fluorometer (Thermo Fisher Scientific). For each sample, 50 ng of genomic DNA was fragmented using the Twist Library Preparation Kit for enzymatic fragmentation (Twist Bioscience, San Francisco, CA, US). Sequencing libraries were prepared using a custom designed target enrichment panel (TE-96631432 from Twist Bioscience).

Pools of sequencing ready libraries from 16 samples were combined in equimolar ratios and sequenced with Mid-Output kits on NextSeq 500/550 sequencers (Illumina, San Diego, CA, US) aiming for a sequencing depth of 750x of 2*150 bp paired-end reads.

**Myeloid panel design**

The design of the custom panel is an extension of the Genomics Medicine Sweden Myeloid panel (GMS-MGP) [Orsmark-Pietras et al. 2024]. In brief the design includes 199 target genes that are either putative driver genes in adult or pediatric myeloid malignancies, of pharmacogenetic relevance (CYP2D6, DPYD, NUDT15, TPMT, UGT1A1), or non-coding genetic regions of interest for inherited hematological malignancies (TERC, TP53 exon 1, FACI1 intron 31, DKC1 5’UTR and exon1 + the 3’UTR of NOTCH1) (Supplementary Table 1). A total target region of 729,001 bp is covered by 7,867 probes. For most target genes, the design covers the coding sequence. The design also includes a backbone of SNVs across the genome for CNV detection and a few miRNAs.

**Bioinformatics processing**

Demultiplexing was performed on instrument using the Local Run Manager v2.4.0 Generate FASTQ Analysis Module (Illumina). Bioinformatic workup of the sequence data was performed using the Pomfrey pipeline (https://github.com/clinical-genomics-uppsala/pomfrey). In brief, the pipeline trim sequence reads using Cutadapt (v2.5) [Martin *et al*.], align sequence reads to the human reference GRCh37.75 using bwa v0.7.17 [Li *et al*.] and deduplicate alignments using Picard MarkDuplicates [http://broadinstitute.github.io/picard]. Coverage across all targeted positions is analyzed using Mosdepth (v0.3.2) [Pedersen *et al*.] and any target below 100x is reported. Small nucleotide variants (SNV) and smaller insertions and deletions (indels) are called using Mutect2 (GATK v4.1.7.0) [Benjamin *et al.*], Pisces (v5.2.11) [Dunn T *et al*.], VarDict (v1.7.0) [Lai *et al*.] and freebayes (v1.3.1) [Garrison *et al*.]. Pindel (v0.2.5b9) [Ye *et al*.] is used to detect larger insertions and deletions, including the *FLT3*-ITD. Copy number variants are detected using CNVkit (v0.9.9) [Talevich *et al*.] and GATK v4.1.7.0 [Pedersen et al.].

SNVs that are detected in at least three of the four callers and all indels from Mutect2 and Vardict are annotated against the canonical transcript in Variant Effect Predictor (v99) [McLaren *et al*.] and common variants (population allele frequency > 2%) and artefacts are filtered. Variants were classified as artefacts if they were detected more than twice in a group of 20 normal samples, or in more than 10% of 128 myeloid samples, unless reported as haematopoietic and lymphoid tissue entries in the COSMIC database v86. Protein coding variants or variants within 6 bp from intron/exon boundaries with allele frequencies above 3% are reported and uploaded for clinical interpretation in QIAGEN Clinical Insight Interpret (QIAGEN). The alignment and annotation of all reported variants were manually inspected in the Integrative Genomics Viewer [Robinson *et al*.].

**References**

- Martin, M. (2011). Cutadapt removes adapter sequences from high-throughput sequencing reads. EMBnet.journal, 17(1), pp. 10-12. doi:<https://doi.org/10.14806/ej.17.1.200>
- Li H, Durbin R. Fast and accurate short read alignment with Burrows–Wheeler transform. *Bioinformatics*. 2009;25(14):1754-1760. doi:10.1093/bioinformatics/btp324
- D Benjamin, T Sato, K Cibulskis, G Getz, C Stewart, L Lichtenstein. Calling Somatic SNVs and Indels with Mutect2. bioRxiv 2019. doi:https://doi.org/10.1101/861054.
- Dunn T, Berry G, Emig-Agius D, Jiang Y, Lei S, Iyer A, Udar N, Chuang HY, Hegarty J, Dickover M, Klotzle B, Robbins J, Bibikova M, Peeters M, Strömberg M. Pisces: an accurate and versatile variant caller for somatic and germline next-generation sequencing data. Bioinformatics. 2019 May 1;35(9):1579-1581. doi: 10.1093/bioinformatics/bty849.
- Lai Z, Markovets A, Ahdesmaki M, Chapman B, Hofmann O, McEwen R, Johnson J, Dougherty B, Barrett JC, Dry JR. VarDict: a novel and versatile variant caller for next-generation sequencing in cancer research. Nucleic Acids Res. 2016 Jun 20;44(11):e108. doi: 10.1093/nar/gkw227. Epub 2016 Apr 7. PMID: 27060149; PMCID: PMC4914105.
- Garrison E, Marth G. Haplotype-based variant detection from short-read sequencing. *arXiv preprint arXiv:1207.3907*, 2012.
- Ye K, Schulz MH, Long Q, Apweiler R, Ning Z. Pindel: a pattern growth approach to detect break points of large deletions and medium sized insertions from paired-end short reads. *Bioinformatics*. 2009;25(21):2865-2871. doi:10.1093/bioinformatics/btp394
- Talevich E, Shain AH, Botton T, Bastian BC. CNVkit: Genome-Wide Copy Number Detection and Visualization from Targeted DNA Sequencing. *PLOS Computational Biology*. 2016;12(4):e1004873. doi:10.1371/journal.pcbi.1004873
- Pedersen BS, Quinlan AR. Mosdepth: quick coverage calculation for genomes and exomes. Bioinformatics. 2018 Mar 1;34(5):867-868. doi: 10.1093/bioinformatics/btx699. PMID: 29096012; PMCID: PMC6030888.
- McLaren W, Gil L, Hunt SE, Riat HS, Ritchie GR, Thormann A, Flicek P, Cunningham F. The Ensembl Variant Effect Predictor. Genome Biol. 2016 Jun 6;17(1):122. doi: 10.1186/s13059-016-0974-4. PMID: 27268795; PMCID: PMC4893825.
- Robinson JT, Thorvaldsdóttir H, Wenger AM, Zehir A, Mesirov JP. Variant Review with the Integrative Genomics Viewer. Cancer Res. 2017 Nov 1;77(21):e31-e34. doi: 10.1158/0008-5472.CAN-17-0337. PMID: 29092934; PMCID: PMC5678989.
