# Supplementary material for: Low Rate of Germline Investigation for Variants of Suspected Germline Origin Detected During the Diagnostic Work‐Up of Myeloid Neoplasms
Source: EJHaem. 2025 Dec 16;6(6):e70206. doi: 10.1002/jha2.70206 (PMC12707176; doi:10.1002/jha2.70206)

Supplementary 3 – Graphs

Figure S3.1

Percentage of investigated patients according to the Nordic guidelines, by suspected myeloid neoplasm and age category

S3.1a) suspected myeloid neoplasm

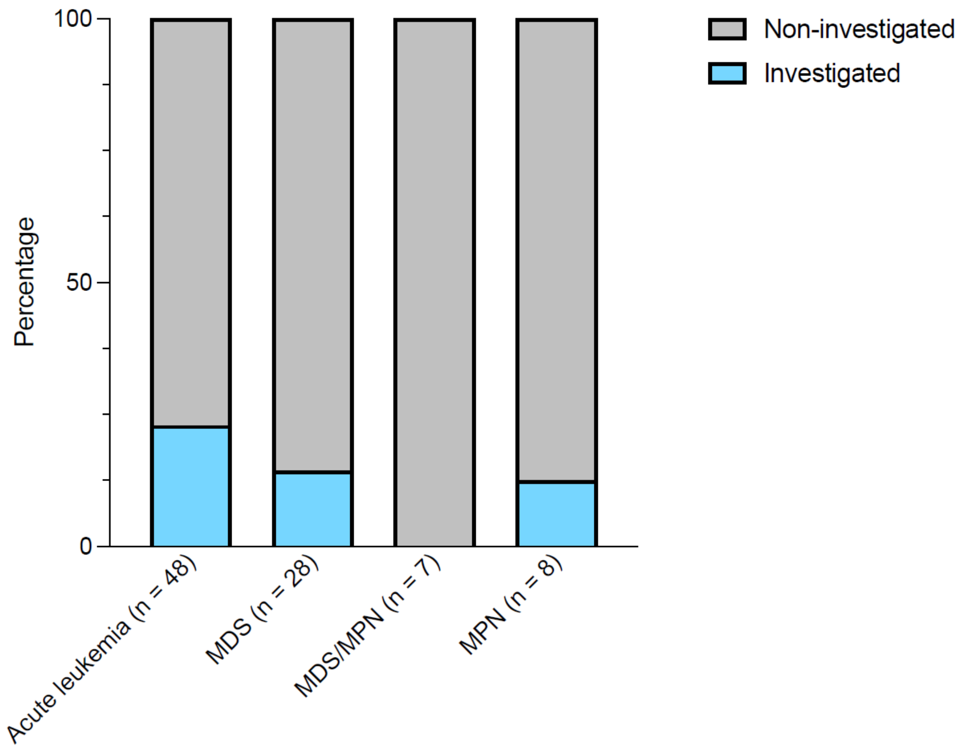

S3.1b) age

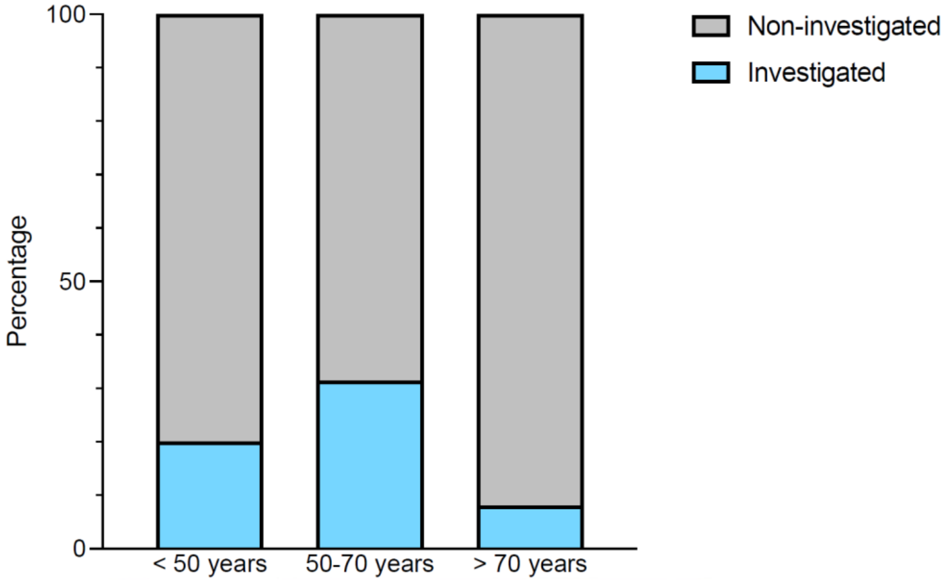

Supplementary 3 – Graphs

Figure S3.2 - The total number of pathogenic and likely pathogenic variants with variant allele frequency between 30-40%, note that one patient might have several unique variants.

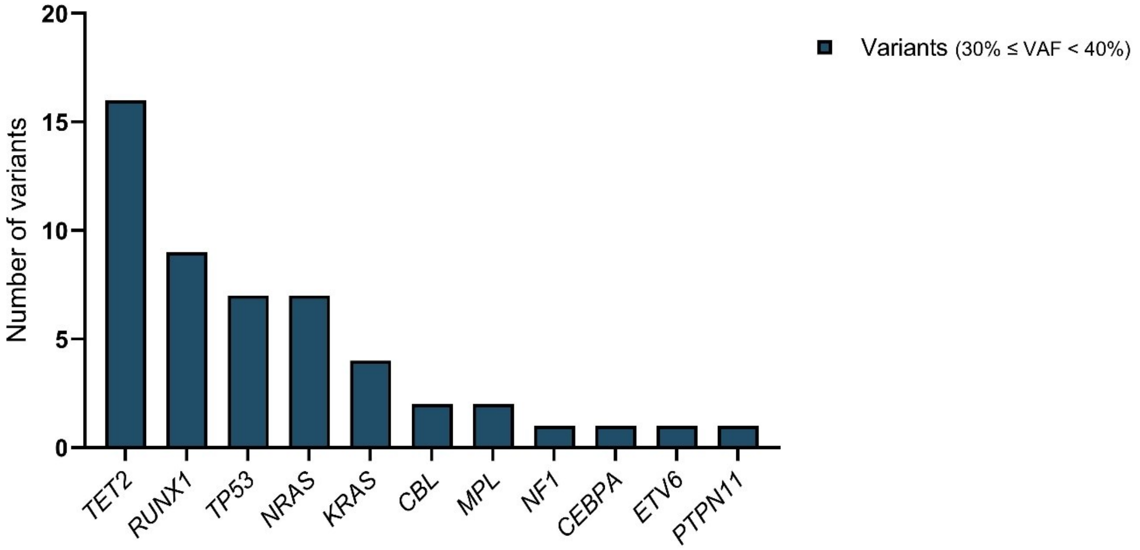

Supplement: Supplementary file 3 — Supporting File 1 [file JHA2-6-e70206-s004.pdf]
